# VEGF inhibitor–induced proteinuria: the role of hypertension and vascular endothelial dysfunction

**DOI:** 10.1186/s40780-026-00586-y

**Published:** 2026-06-25

**Authors:** Satoru Nihei, Kazuki Saito, Kenzo Kudo

**Affiliations:** 1 Department of Pharmacy, Iwate Medical University Hospital, 2-1-1 Idaidori, Yahaba-cho, Shiwa-gun, Iwate, 028-3695 Japan; 2 Division of Clinical Pharmaceutics and Pharmacy Practice, Department of Clinical Pharmacy, School of Pharmacy, Iwate Medical University, 1-1-1 Idaidori, Yahaba-cho, Shiwa-gun, Iwate 028-3694 Japan

**Keywords:** Vascular endothelial growth factor (VEGF) inhibitors, Proteinuria, Hypertension, Vascular endothelial dysfunction, Nitric oxide, Endothelin-1

## Abstract

Vascular endothelial growth factor (VEGF) inhibitors are widely used anticancer agents that suppress tumor angiogenesis and improve outcomes in various solid tumors. However, these therapies are frequently associated with adverse renal and vascular events, particularly hypertension and proteinuria, which may compromise treatment continuity and patient quality of life. Although hypertension has long been considered a major contributor to VEGF inhibitor–induced proteinuria, the precise relationship between these complications remains incompletely understood. In addition, accumulating evidence suggests that endothelial dysfunction—characterized by impaired nitric oxide bioavailability, increased endothelin-1 signaling, and microvascular injury—plays a central role in the pathogenesis of these adverse events, potentially linking hypertension and proteinuria through a shared underlying mechanism. This review summarizes current evidence regarding the role of blood pressure and vascular endothelial dysfunction in the development of proteinuria during VEGF inhibitor therapy. Multiple clinical studies have demonstrated that both baseline and on-treatment blood pressure are associated with an increased risk of proteinuria. However, the causal relationship between hypertension and proteinuria remains uncertain. Hypertension may contribute to glomerular injury by increasing intraglomerular pressure, but it may also represent a parallel manifestation of endothelial injury caused by VEGF signaling blockade. Emerging evidence suggests that vascular endothelial dysfunction plays a key role in this process. Studies using flow-mediated dilation and reactive hyperemia index measurements have demonstrated impaired endothelial function during VEGF inhibitor therapy. In addition, alterations in endothelial-derived vasoactive mediators, including reduced nitric oxide bioavailability and increased endothelin-1 signaling, have been reported. Taken together, VEGF inhibitor–induced proteinuria likely results from complex interactions between systemic hypertension, endothelial dysfunction, and glomerular microvascular injury. Further translational and prospective clinical studies are needed to clarify the causal mechanisms and optimize strategies for prediction, prevention, and management of this complication.

## Background

Recent cancer therapeutic agents targeting the vascular endothelial growth factor (VEGF) signaling pathway include bevacizumab, a monoclonal antibody that neutralizes VEGF-A, ramucirumab, a monoclonal antibody targeting VEGF receptor 2 (VEGFR2), and small-molecule inhibitors known as VEGF receptor tyrosine kinase inhibitors (TKIs), such as sunitinib and sorafenib. These agents suppress tumor angiogenesis and are widely used in the treatment of various solid tumors, including colorectal cancer, non-small cell lung cancer, hepatocellular carcinoma, and renal cell carcinoma [1–3].

However, the use of VEGF inhibitors is associated with several renal and vascular adverse events due to inhibition of VEGF signaling [4–6]. Among these toxicities, hypertension and proteinuria are particularly common, and may be severe enough to require dose reduction, treatment interruption, or discontinuation of therapy, potentially affecting patient prognosis and quality of life [7–9]. Therefore, appropriate understanding and management of these adverse events are essential for maintaining the continuity and safety of cancer treatment.

VEGF inhibitor–induced proteinuria is thought to arise because glomerular endothelial cell function and the integrity of the glomerular filtration barrier depend on signaling mediated by podocyte-derived VEGF-A through endothelial VEGF receptor 2 [10, 11]. Inhibition of VEGF signaling might damage glomerular endothelial cells and disrupt the filtration barrier, leading to proteinuria; however, the detailed molecular mechanisms of this effect remain incompletely understood [12].

Hypertension and proteinuria frequently develop concurrently during VEGF inhibitor therapy, suggesting shared underlying mechanisms. VEGF inhibition induces hypertension through dysregulation of vascular tone and endothelial homeostasis, while also affecting renal microcirculation [13, 14]. These observations raise the possibility that both conditions reflect a common vascular injury process rather than a simple unidirectional cause–effect relationship. Importantly, the relationship between hypertension and proteinuria in this setting is conceptually complex. Hypertension may act not only as a causal mediator of glomerular injury through increased intraglomerular pressure, but also as a clinical marker or parallel manifestation of VEGF inhibitor–induced endothelial dysfunction.

To better conceptualize this complexity, VEGF inhibitor–induced proteinuria can be understood as arising from three interrelated mechanisms: (1) hemodynamic injury driven by systemic and intraglomerular hypertension, (2) blood pressure–independent endothelial injury characterized by thrombotic microangiopathy–like changes, and (3) shared upstream endothelial dysfunction involving imbalance of vasoactive mediators such as nitric oxide (NO) and endothelin-1 (ET-1). This framework provides a basis for integrating clinical observations with mechanistic insights and may help refine risk stratification and management strategies.

Although previous reviews have addressed hypertension and proteinuria associated with VEGF inhibitors, these complications have largely been discussed separately, and their interplay remains incompletely understood. In particular, while endothelial dysfunction is widely recognized as a key contributor to proteinuria, its clinical significance in VEGF inhibitor–associated toxicity has not been fully established. It therefore remains unclear whether hypertension and endothelial dysfunction act as causal factors, predictive markers, or parallel manifestations of VEGF inhibitor–induced vascular injury.

In this review, we synthesize clinical, translational, and experimental evidence to clarify the relationship between blood pressure, endothelial dysfunction, and proteinuria in patients receiving VEGF inhibitors. We further propose an integrated conceptual framework to better understand the underlying pathophysiology and to identify key knowledge gaps relevant to clinical management.

## Role of hypertension in VEGF inhibitor–induced proteinuria

Blood pressure control plays a critical role in the development and progression of proteinuria in many kidney diseases, including diabetic and non-diabetic kidney disease [15–17]. Hypertension is also one of the most common adverse events in patients receiving VEGF inhibitors, with many patients experiencing increases in blood pressure during treatment. Several clinical studies, most of which were retrospective, have reported associations between baseline and on-treatment blood pressure and the development of proteinuria in patients receiving VEGF inhibitors (Table 1) [18–35].

**Table 1:** Review of studies on hypertension-related factors associated with VEGF inhibitor–induced proteinuria

Year	First author	VEGF inhibitor(s)	Population/n	Definition of proteinuria outcome	Significant baseline hypertension-related factors	Significant on-treatment hypertension-related factors
2014	Baek [18]	Sunitinib	Multiple solid tumors/155	Any grade	SBP/DBP ≥ 140/90 mmHg ↑	NR
2016	Sorich [19]	Pazopanib, Sunitinib	Renal cell carcinoma/1,392	Any grade; Grade 3–4	SBP (per 10 mmHg) ↑	NR
2017	Boissier [20]	Multiple VEGF inhibitors	Multiple solid tumors/168	Any grade	None	Grade ≥ 2 hypertension ↑
2018	Nihei [21]	Bevacizumab	Lung cancer/211	Any grade	SBP/DBP ≥ 140/90 mmHg ↑; RASI use ↓	NR
2019	Hirai [22]	Bevacizumab, Ramucirumab	Solid tumors/208	Grade ≥ 2	RASI use ↓	NR
2020	Cappagli [23]	Cabozantinib	Thyroid cancer/18	Any grade	None	NR
2020	Kanbayashi [24]	Bevacizumab, Ramucirumab, Aflibercept	Colorectal, gastric, breast, and lung cancer/139	Any grade	SBP (continuous) ↑; CCB use ↑	NR
2021	Shimamura [25]	Lenvatinib	Hepatocellular carcinoma/45	Nephrotic-range proteinuria	None	NR
2022	Kimura [26]	Ramucirumab	Multiple solid tumors/145	Any grade; Grade ≥ 2	None	NR
2022	Ikesue [27]	Lenvatinib	Hepatocellular carcinoma/37	Grade ≥ 2	None	NR
2022	Ikesue [28]	Axitinib	Renal cell carcinoma/50	Any grade; Grade ≥ 2	Non-RASI antihypertensive drugs use ↑	NR
2022	Ando [29]	Bevacizumab	Hepatocellular carcinoma/64	Any grade; PCR ≥ 2 g/gCr	Treatment for hypertension ↑; SBP ≥ 130 mmHg ↑	Average SBP ≥ 135 mmHg ↑
2022	Chiba [30]	Ramucirumab	Gastric cancer/36	Any grade	RASI use ↓	NR
2023	Kiyomi [31]	Bevacizumab	Solid tumors/2,458	Any grade	SBP ≥ 140 mmHg ↑	NR
2024	Nihei [32]	Bevacizumab	Colorectal cancer/279	Grade ≥ 2; PCR ≥ 2 g/gCr	None	Average SBP ≥ 140 mmHg ↑
2024	Saito [33]	Regorafenib	Colorectal cancer/100	Any grade	None	NR
2024	Hanna [34]	Multiple VEGF inhibitors	Solid tumors/1,249	Grade ≥ 2	SBP ≥ 160 mmHg ↑	NR
2024	Yang [35]	Bevacizumab, Lenvatinib	Hepatocellular carcinoma/622	Grade ≥ 2	History of hypertension ↑	NR

↑: statistically significant positive association with the risk of proteinuria; ↓: statistically significant inverse association with the risk of proteinuria

None: no significant association was identified; NR: the factor was not evaluated or not reported

SBP: systolic blood pressure; DBP: diastolic blood pressure; RASI: renin–angiotensin system inhibitor; CCB: calcium channel blocker; PCR: protein-to-creatinine ratio

Baseline blood pressure appears to be an important predictor of proteinuria. In a study of patients treated with bevacizumab, baseline systolic blood pressure (SBP)/diastolic blood pressure (DBP) ≥140/90 mmHg was significantly associated with the development of proteinuria [21]. Similar findings were reported by Baek et al. in patients treated with sunitinib [18]. Other studies have also highlighted the importance of SBP in predicting proteinuria [19, 24, 29, 31, 34]. Although different cutoff values have been used across studies (e. g. , SBP ≥ 130, ≥140, and ≥160 mmHg), an increased risk of proteinuria has been consistently observed with higher blood pressure levels. In contrast, findings regarding the influence of a history of hypertension itself have been inconsistent. This inconsistency may reflect differences in blood pressure control, as well-controlled hypertension might not confer an increased risk. In addition, comorbid conditions such as diabetes mellitus, chronic kidney disease, and dyslipidemia may act as confounding factors, potentially obscuring the independent effect of hypertension.

On-treatment blood pressure has also been associated with the development of proteinuria. Ando et al. reported a high incidence of severe proteinuria in patients with a mean SBP ≥ 135 mmHg during bevacizumab therapy [29]. In our previous study, a mean SBP ≥ 140 mmHg during the first three months of treatment was associated with a higher subsequent risk of proteinuria [32]. These findings suggest that early blood pressure trends during VEGF inhibitor therapy may represent an important determinant of proteinuria risk.

However, the causal relationship between hypertension and proteinuria during VEGF inhibitor therapy remains unclear. Elevated systemic blood pressure may increase intraglomerular pressure and promote glomerular injury. Alternatively, hypertension and proteinuria may represent parallel manifestations of VEGF inhibitor–induced endothelial dysfunction, in which VEGF signaling blockade causes endothelial injury that simultaneously leads to systemic hypertension and glomerular microvascular damage. A third possibility is that hypertension primarily reflects on-target VEGF inhibition and thus serves as a clinical marker of VEGF inhibitor–associated vascular toxicity, rather than directly contributing to the development of proteinuria. These mechanisms are not mutually exclusive and may all contribute to proteinuria to varying degrees.

Establishing appropriate blood pressure management targets during VEGF inhibitor therapy remains challenging. In general hypertension management, a target blood pressure of <130/80 mmHg is recommended [36], and this target is generally considered applicable to patients with cancer. In patients undergoing cancer treatment, antihypertensive therapy is typically initiated at a blood pressure of 140/90 mmHg, while earlier intervention may be considered in those with comorbid cardiovascular disease, chronic kidney disease, or diabetes [37]. In clinical practice, target blood pressure is often initially set at <140/90 mmHg and, if tolerated, further reduced to <130/80 mmHg. In patients with metastatic cancer, however, slightly higher blood pressure levels (approximately 140–160/90–100 mmHg) may be acceptable, depending on overall prognosis and treatment priorities [38, 39].

Renin–angiotensin system inhibitors (RASIs), including ACE inhibitors and angiotensin II receptor blockers, are recommended as first-line agents for hypertension accompanied by proteinuria [36, 40]. However, evidence supporting their specific benefit in VEGF inhibitor–induced proteinuria remains limited. Several retrospective studies have reported that the use of non-RASI antihypertensive agents, including calcium channel blockers (CCBs), was associated with a higher incidence of proteinuria [24, 28], whereas RASIs may potentially reduce this risk [21, 22, 30]. However, these findings should be interpreted cautiously because retrospective analyses are susceptible to differences in blood pressure control, treatment selection bias, and residual confounding. In particular, CCBs may have been more frequently prescribed to patients with higher blood pressure levels, and this potential confounding should be considered when interpreting these associations. In a small randomized trial involving approximately 30 patients comparing azilsartan and amlodipine for bevacizumab-induced hypertension and proteinuria, no significant difference in proteinuria was observed between the two treatments [41]. These findings suggest that the overall quality of blood pressure control may be more important than the specific class of antihypertensive agent used.

Accordingly, blood pressure control—particularly during the early phase of treatment—appears to be a clinically modifiable factor associated with the development of proteinuria during VEGF inhibitor therapy. Continuous blood pressure monitoring and appropriate management throughout treatment may therefore help reduce the risk of VEGF inhibitor–induced proteinuria. In addition, multidisciplinary collaboration among oncologists, nephrologists, nurses, and pharmacists may facilitate earlier detection and intervention. Because increases in blood pressure may precede clinically significant proteinuria, proactive monitoring during treatment may provide an opportunity for earlier intervention.

However, these observational findings should be interpreted with caution because most available evidence is derived from retrospective analyses and is therefore susceptible to confounding by indication, treatment selection bias, and time-dependent confounding. For example, patients with higher blood pressure are more likely to receive antihypertensive therapy, which may influence both blood pressure trajectories and proteinuria outcomes. In addition, reverse causality cannot be excluded, as the development of proteinuria may itself lead to intensification of antihypertensive treatment.

Interpretation of the current literature is further complicated by substantial heterogeneity in the definition and assessment of proteinuria across studies. Previous reports have variably used dipstick testing, the Common Terminology Criteria for Adverse Events (CTCAE) grading, urinary protein-to-creatinine ratio (PCR), and nephrotic-range proteinuria as study endpoints. Dipstick testing is a qualitative assessment and, together with CTCAE grading based on dipstick results, may not adequately reflect the severity or persistence of urinary protein excretion. Although CTCAE criteria also incorporate 24-hour urinary protein excretion, such measurements are infrequently performed in routine clinical practice. In contrast, PCR, which can be measured using spot urine samples, provides a practical surrogate for quantitative assessment of 24-hour urinary protein excretion. Importantly, transient low-grade proteinuria may differ substantially in clinical significance from persistent or nephrotic-range proteinuria associated with structural glomerular injury. This heterogeneity limits direct comparison among studies and complicates interpretation of the available evidence.

Taken together, these limitations preclude definitive conclusions regarding causality. Prospective studies with longitudinal assessment of blood pressure and proteinuria outcomes, together with analytical approaches that account for time-varying confounders, are needed to better clarify the causal role of hypertension in VEGF inhibitor–induced proteinuria.

## Role of vascular endothelial dysfunction in VEGF inhibitor–induced proteinuria

Proteinuria associated with VEGF inhibitor therapy cannot be explained solely by elevated blood pressure, and accumulating evidence supports a central role for vascular endothelial dysfunction as a shared upstream mechanism. From a pathophysiological perspective, VEGF signaling inhibition disrupts endothelial homeostasis, leading to reduced NO bioavailability, decreased prostacyclin activity, and increased ET-1 signaling. This imbalance promotes vasoconstriction, microvascular rarefaction, and increased vascular resistance, ultimately resulting in both systemic hypertension and glomerular microvascular injury.

Within the kidney, these processes may induce endothelial swelling, loss of fenestrations, and thrombotic microangiopathy–like lesions, which can occur even in the absence of severe systemic hypertension. Endothelial dysfunction, characterized by impaired vasodilation, enhanced inflammatory signaling, and a prothrombotic state, is widely implicated in cardiovascular and renal diseases [42]. Taken together, these observations support the concept that endothelial injury represents a key driver of VEGF inhibitor–induced proteinuria, at least in part independent of systemic hemodynamic effects.

Several studies have evaluated endothelial function in patients receiving VEGF inhibitors (Table 2) [43–51]. Noninvasive methods, such as flow-mediated dilation (FMD) and the reactive hyperemia index (RHI), are commonly used to assess endothelial function. Steeghs et al. reported decreased FMD and increased blood pressure several weeks after initiation of VEGF inhibitor therapy [44, 45]. Sueta et al. observed early reductions in RHI shortly after initiation of lenvatinib therapy [49]. Similarly, Lai et al. reported decreased FMD accompanied by increases in blood pressure and proteinuria during sunitinib treatment [50].

**Table 2:** Clinical studies on endothelial dysfunction associated with VEGF inhibitor therapy

Year	First author	VEGF inhibitor(s)	Population/n	Timing of measurement	Endothelial function findings	Proteinuria findings	Blood pressure findings
2008	Mourad [43]	Bevacizumab	Colorectal cancer/18	Baseline, 6 months	Forearm skin perfusion decreased	NR	SBP and DBP increased
2008	Steeghs [44]	Telatinib	Multiple solid tumors/18	Baseline, Week 5	FMD decreased	Proteinuria reported	SBP and DBP increased
2010	Steeghs [45]	Bevacizumab	Breast and colorectal cancer/14	Baseline, Week 6, >3 months after discontinuation	FMD decreased	NR	DBP increased
2010	van der Veldt [46]	Sunitinib	Renal cell carcinoma/16	Baseline, Day 14, Day 28	Laser Doppler flowmetry unchanged	NR	SBP and DBP increased
2011	Mayer [47]	Vandetanib	Breast cancer/17	Baseline, Week 6	FMD unchanged	NR	SBP and DBP increased
2015	Thijs [48]	Sunitinib	Renal cell carcinoma/10	Baseline, Week 1 (BP monitored through Week 2)	Acetylcholine-induced vasorelaxation unchanged	NR	Mean arterial pressure increased
2017	Sueta [49]	Lenvatinib	Thyroid cancer/10	Baseline, Days 1–6	RHI decreased	NR	SBP increased
2020	Lai [50]	Sunitinib	Renal cell carcinoma/20	Baseline, Day 40	FMD decreased	Proteinuria increased	SBP and DBP increased
2025	Nihei [51]	Bevacizumab	Hepatocellular carcinoma/20	Baseline, 3 months, 6 months	RHI decreased	PCR associated with RHI	SBP associated with RHI

NR:the factor was not evaluated or not reported

FMD: flow-mediated dilation; SBP: systolic blood pressure; DBP: diastolic blood pressure; RHI: reactive hyperemia index; PCR: protein-to-creatinine ratio

Consistent with these findings, our previous prospective study of patients treated with bevacizumab demonstrated a reduction in RHI during treatment, and a significant association between RHI and both urinary protein-to-creatinine ratio and systolic blood pressure [51]. These findings support the concept that endothelial dysfunction may contribute to the development of both hypertension and proteinuria during VEGF inhibitor therapy. Notably, some studies have suggested that TKIs may induce endothelial dysfunction earlier than bevacizumab. Although this difference may partly reflect variations in the timing of endothelial function assessment across studies, class-specific differences among VEGF inhibitors may also influence the onset and progression of endothelial dysfunction, as well as the subsequent development of hypertension and proteinuria. However, the available evidence should be interpreted cautiously because most previous studies involved relatively small cohorts and relied on surrogate markers of endothelial function; therefore, causal relationships remain uncertain.

An imbalance in endothelial-derived vasoactive mediators has been proposed as a key mechanism underlying these vascular changes. Reduced NO bioavailability, decreased prostacyclin activity, and increased endothelin signaling have each been implicated, at least in part, in VEGF inhibitor–associated vascular toxicity (Table 3) [47, 49, 52–55]. Clinical studies have reported decreased NO metabolites during VEGF inhibitor therapy. Robinson et al. observed reduced urinary nitrate/nitrite (NOx) levels in patients treated with VEGF inhibitors [52], while Mayer et al. and Sueta et al. reported decreases in plasma or serum NOx, accompanied by increases in blood pressure, during treatment with vandetanib and lenvatinib, respectively [47, 49]. Tinning et al. further demonstrated an association between plasma NOx levels and systolic blood pressure in patients receiving pazopanib [53]. These findings suggest that reduced NO bioavailability may contribute to the development of VEGF inhibitor–related vascular dysfunction.

**Table 3:** Clinical studies investigating endothelial dysfunction pathways associated with VEGF inhibitor–induced hypertension and proteinuria

Year	First author	VEGF inhibitor(s)	Population/n	Measurement timing	Endothelium-derived vasoactive mediators	Proteinuria findings	Blood pressure findings
2010	Robinson [52]	Multiple VEGF inhibitors	Renal cell carcinoma/80 (VEGF inhibitor *n* = 40; control *n* = 40)	On-treatment (VEGFi vs. control)	Decreasing trend in urinary NOx/Cr; 6-keto PGF1α/Cr unchanged	ACR was not associated with urinary NOx/Cr or 6-keto PGF1α/Cr	SBP unchanged; increased use of antihypertensive medications
2011	Mayer [47]	Vandetanib	Breast cancer/17	Baseline, Week 6	Plasma NOx decreased	NR	SBP and DBP increased
2017	Sueta [49]	Lenvatinib	Thyroid cancer/10	Baseline, Days 1–6	Serum NOx decreased	NR	SBP increased
2018	Tinning [53]	Pazopanib	Renal cell carcinoma/27	Baseline, Week 4	Urinary NOx/Cr decreased, whereas ET-1/Cr remained unchanged. Plasma NOx decreased and ET-1 increased	PCR was not associated with urinary or plasma NOx or ET-1	SBP was associated with plasma NOx
2010	Kappers [54]	Sunitinib	Renal cell carcinoma and gastrointestinal stromal tumor/15	Baseline, Week 4, Week 10	Plasma ET-1 increased	NR	SBP and DBP increased
2023	Nihei [55]	Bevacizumab	Colorectal cancer/40	Baseline, 3 months, 6 months	Plasma ET-1 increased	Grade ≥ 2 proteinuria associated with plasma ET-1	NR

NR:the factor was not evaluated or not reported

NOx: nitrate/nitrite; Cr: creatinine; PG: prostacyclin; ACR: albumin-to-creatinine ratio; SBP: systolic blood pressure; DBP: diastolic blood pressure; ET-1: endothelin-1; PCR: protein-to-creatinine ratio

Activation of the endothelin-1 (ET-1) pathway represents another potential mechanism underlying VEGF inhibitor–associated toxicity. Kappers et al. reported increased plasma ET-1 levels during sunitinib therapy [54], and we observed that elevated plasma ET-1 levels in patients receiving bevacizumab were associated with Grade ≥ 2 proteinuria [55]. Mechanistic studies further support a causal role of endothelin signaling. In vitro, bevacizumab exposure increased ET-1 levels in both intracellular compartments and culture supernatants of human glomerular microvascular endothelial cells, suggesting that this effect may be mediated through VEGF-A signaling [56]. In vivo, endothelin receptor antagonists attenuated hypertension and proteinuria in a sunitinib-induced nephropathy rat model, providing functional evidence that endothelin contributes directly to VEGF inhibitor–associated renal injury [57, 58]. Together, these findings suggest that dysregulation of endothelial-derived vasoactive mediators contributes to both systemic hypertension and renal vascular injury.

Renal pathological findings provide further evidence that VEGF inhibitor–associated proteinuria cannot be explained solely by systemic hypertension. Renal biopsy studies have demonstrated characteristic lesions, including thrombotic microangiopathy (TMA), glomerular endotheliosis, and other forms of endothelial injury in patients treated with anti-VEGF agents [13]. These changes are characterized by endothelial cell swelling, loss of fenestrations, subendothelial expansion, and microvascular thrombosis within glomerular capillaries. Notably, the occurrence of TMA-like lesions even in the absence of severe systemic hypertension suggests that glomerular endothelial injury may develop independently of blood pressure elevation. Emerging evidence also suggests that the pathological manifestations and underlying mechanisms of renal injury may differ among VEGF pathway inhibitors, including anti-VEGF antibodies, anti-VEGFR2 antibodies, VEGF traps, and TKIs. In particular, TMA has been reported predominantly with anti-VEGF ligand therapies, whereas minimal change nephropathy and collapsing focal segmental glomerulosclerosis-like lesions have been observed mainly with TKIs, suggesting distinct patterns of endothelial and podocyte injury depending on the class of VEGF pathway inhibition [13]. These observations underscore the critical role of VEGF signaling in maintaining glomerular endothelial integrity and provide direct pathological evidence linking VEGF inhibition to endothelial injury.

Taken together, these findings support a model in which VEGF inhibitor–induced proteinuria arises from a cascade of vascular injury: VEGF signaling blockade induces endothelial dysfunction, disrupts the balance of vasoactive mediators, and ultimately leads to structural injury of the glomerular endothelium. However, much of the available evidence is derived from small clinical studies using surrogate markers of endothelial function or from experimental models. Thus, although endothelial dysfunction provides a coherent mechanistic framework linking VEGF inhibition, hypertension, and renal injury, definitive causal relationships remain to be established.

An important limitation of current clinical evidence is that most assessments of endothelial function rely on systemic surrogate markers, such as flow-mediated dilation and circulating biomarkers, which may not accurately reflect the renal microenvironment. The kidney possesses a unique microvascular architecture and is exposed to distinct hemodynamic and paracrine conditions; therefore, systemic endothelial markers may not adequately capture local glomerular injury. In addition, many clinical studies evaluating endothelial dysfunction during VEGF inhibitor therapy have relatively short observation periods, often limited to several weeks, potentially limiting assessment of cumulative endothelial injury. Severe proteinuria may develop during later phases of treatment in some patients, suggesting that VEGF inhibitor–induced proteinuria may exhibit a temporal pattern distinct from that of blood pressure elevation and may reflect cumulative endothelial injury [32, 59]. Future studies incorporating urinary biomarkers, renal-specific endothelial markers, and direct pathological evaluation are needed to better characterize the renal endothelial response to VEGF inhibition. These limitations may partly explain the inconsistency between systemic endothelial markers and the severity of proteinuria observed in clinical practice.

## Conclusion

VEGF inhibitor–induced proteinuria is an important adverse event that may compromise the continuity of cancer treatment. Both hypertension and endothelial dysfunction appear to contribute to its development. Clinical evidence consistently indicates that both baseline and on-treatment hypertension are associated with an increased risk of proteinuria, suggesting that blood pressure control represents a clinically modifiable factor for mitigating renal injury during VEGF inhibitor therapy. In current clinical practice, management strategies to reduce the risk of proteinuria should include not only dose interruption or discontinuation of VEGF inhibitors when necessary, but also appropriate blood pressure monitoring and multidisciplinary management.

However, VEGF inhibitor–induced proteinuria cannot be explained solely by elevated blood pressure. Increasing evidence supports a central role for endothelial dysfunction and imbalance of vasoactive mediators, including reduced NO bioavailability and enhanced endothelin signaling, in VEGF inhibitor–associated vascular toxicity. Importantly, hypertension in this setting may represent not only a potential causative factor but also a clinical marker or parallel manifestation of VEGF inhibitor–induced endothelial injury. Despite these insights, the optimal blood pressure targets and the most effective antihypertensive strategies during VEGF inhibitor therapy remain to be established. In addition, systemic endothelial markers may not fully capture the renal microenvironment.

From a clinical perspective, these findings suggest that management strategies for VEGF inhibitor–induced proteinuria should not focus solely on absolute blood pressure thresholds, but also consider early blood pressure trajectories and underlying endothelial dysfunction. Blood pressure control remains a key modifiable factor, particularly during the early phase of treatment; however, optimal target levels may need to be individualized based on patient characteristics, comorbidities, and treatment intent.

Furthermore, the potential role of endothelial dysfunction as a therapeutic target warrants further investigation. Strategies aimed at restoring endothelial homeostasis, in addition to conventional antihypertensive therapy, may represent a promising approach for mitigating VEGF inhibitor–associated renal toxicity. Future studies integrating clinical, mechanistic, and pathological approaches will be essential to advance precision management in this setting.

## Data Availability

No datasets were generated or analysed during the current study.

## Abbreviations

CCBs: Calcium channel blockers
CTCAE: Common Terminology Criteria for Adverse Events
DBP: Diastolic blood pressure
ET-1: Endothelin-1
FMD: Flow-mediated dilation
NO: Nitric oxide
NOx: Nitrate/nitrite
PCR: Protein-to-creatinine ratio
RASIs: Renin–angiotensin system inhibitors
RHI: Reactive hyperemia index
SBP: Systolic blood pressure
TKIs: Tyrosine kinase inhibitors
TMA: Thrombotic microangiopathy
VEGF: Vascular endothelial growth factor
VEGFR2: Vascular endothelial growth factor receptor 2
